# Gut Microbiota and Liver Metabolism Regulation Mediate the Protective Effects of Inactivated Selenium-Enriched Yeast Against Alcohol-Induced Liver Damage in Mice

**DOI:** 10.3390/foods14244209

**Published:** 2025-12-08

**Authors:** Zihua Liang, Xiangchen Zhang, Shiwei Chen, Meiting Wang, Deying Men, Wangxin Liu, Xucong Lv

**Affiliations:** 1 Institute of Food Science and Technology, College of Biological Science and Technology, Fuzhou University, Fuzhou 350108, China; 2500810007@fzu.edu.cn (Z.L.); 228527202@fzu.edu.cn (X.Z.); 228527170@fzu.edu.cn (S.C.); 2500810011@fzu.edu.cn (M.W.); 240410034@fzu.edu.cn (D.M.); liuwangxin@fzu.edu.cn (W.L.); 2 Food Nutrition and Health Research Center, School of Advanced Manufacturing, Fuzhou University, Jinjiang 362200, China

**Keywords:** selenium-enriched yeast, alcohol-induced liver damage, protective effects, gut microbiota, liver metabolism, functional food ingredient

## Abstract

Inactivated Selenium-enriched yeast (YSe), as an organic source of selenium with multiple physiological activities, has attracted widespread attention. However, its potential to alleviate alcoholic liver injury (ALD) and its underlying mechanisms remain largely unexplored. This study explores the protective effects of inactivated YSe intervention on ALD in mice and clarifies its mechanism of action. The results indicated that, at the same selenium dose, inactivated YSe intervention was superior to inorganic selenium (sodium selenite) in alleviating ALD. Specifically, high-dose inactivated YSe significantly reduced the levels of serum ALT and AST in alcohol-exposed mice (38.69% and 24.67%, respectively), increased the level of HDL-C (16.83%), and effectively improved alcohol-induced lipid metabolism disorders and liver oxidative damage. At the same time, it significantly increased the concentration of short-chain fatty acids (SCFAs) in feces. 16S rRNA sequencing indicates that inactivated YSe intervention enhances the abundance of beneficial flora (such as *Blautia*, *Oscillibacter*, *Anaerotruncus*, *Butyricicoccus*, and *Ruminiclostridium*) and simultaneously inhibits potentially harmful microbiota (such as *xylanophium*, *Escherichia–Shigella* and *oscilliumspirates*) to restore the homeostasis of the intestinal microbiota in ALD mice. Liver metabolomics analysis revealed that inactivated YSe intervention significantly altered the liver metabolic profile. The core pathways that are regulated by YSe after alcohol disruption include glutathione metabolism, purine metabolism, riboflavin metabolism, etc. In conclusion, this study demonstrates that inactivated YSe can effectively alleviate ALD in mice by regulating the structure of the intestinal flora and restoring liver metabolic homeostasis, providing a scientific basis for its potential functional food component in the prevention and auxiliary management of ALD.

## 1. Introduction

Excessive alcohol consumption represents a significant global public health burden, accounting for approximately 5.3% of global deaths and 5.1% of the disease burden worldwide [1,2]. Following ingestion, alcohol is rapidly absorbed via the gastrointestinal tract into the systemic circulation, The liver, being the primary organ for alcohol metabolism and detoxification [3], is consequently highly susceptible to injury. Chronic alcohol consumption disrupts intestinal barrier integrity, facilitating the translocation of gut-derived harmful substances (e.g., endotoxins) into the portal circulation, thereby exacerbating hepatic damage through inflammatory cascades [4,5]. Concurrently, it promotes hepatic steatosis (characterized by triglyceride accumulation) and systemic dysregulation of lipid metabolism [6]. While pharmacological agents exist for managing alcohol-induced liver disease (ALD), their long-term application is frequently associated with adverse effects, including immunosuppression and heightened infection susceptibility, underscoring the urgent need for safer, natural alternatives with hepatoprotective properties.

Selenium (Se) is an indispensable trace element for human health, serving as a core cofactor for various antioxidant enzymes such as glutathione peroxidase and thioredoxin reductase. It plays a critical role in mitigating oxidative stress, regulating immune responses, and maintaining energy homeostasis [7,8,9]. Clinical evidence indicates that selenium deficiency reduces selenoprotein activity and exacerbates hepatic lipid peroxidation in individuals with chronic alcohol consumption [10]. Selenium exists primarily in organic and inorganic forms in nature. Compared to inorganic forms like selenite and selenate, organic selenium mainly exists as selenomethionine (Se-Met), which is efficiently absorbed via intestinal methionine transporters and can be nonspecifically incorporated into body proteins during protein synthesis, forming a slow-release “selenium reservoir.” This enables sustained support for the synthesis and activity of selenoenzymes, representing the molecular basis for its superior antioxidant efficiency [11,12]. Consequently, organic selenium has garnered significant attention in nutritional interventions and clinical applications due to its higher bioavailability. Although selenium-enriched crops are an important dietary source, their production is constrained by long growth cycles and geographical limitations. In contrast, microbial biotransformation of selenium, particularly yeast fermentation, offers distinct advantages such as low cost, rapid biomass production, and high conversion efficiency, enabling large-scale production of organic selenium-rich biological products [13].

*Saccharomyces cerevisiae* has been confirmed to fall within the category of probiotics, providing health benefits to the host, including the promotion of intestinal health, when administered in adequate amounts [14]. More importantly, specific yeast strains can efficiently absorb inorganic selenium and convert it into organic selenium compounds. The resulting selenium-enriched yeast (YSe) not only possesses intrinsic antioxidant activity but also serves as a key precursor for the synthesis of selenoproteins, which are indispensable in vital processes such as cellular repair and redox homeostasis [15]. However, live Se-enriched yeast carries potential biosafety risks and poor storage stability [16]. To address these limitations, this study utilizes heat-inactivated Se-enriched yeast (YSe). The thermal inactivation process completely eliminates the risk of microbial translocation, extends shelf life, and disrupts the cell wall, promoting the release of organic selenium, polysaccharides, peptides, and other active substances, thereby enhancing absorption rate and bioavailability. The resulting inactivated YSe does not rely on microbial viability but can activate host selenoprotein expression and inhibit hepatic inflammation and lipid peroxidation through a “postbiotic” pathway [17]. This offers a safer, more stable, and mechanistically defined novel strategy for the intervention of ALD, while also broadening the application prospects of Se-enriched functional ingredients in special populations.

Building upon this background, the present study utilized *Saccharomyces cerevisiae* FZU301, previously isolated from *Hongqu* rice wine fermentation, to prepare inactivated YSe, which we define as a selenium-enriched postbiotic. We systematically evaluated the protective effects of this inactivated YSe against ALD in a mouse model. To elucidate the underlying mechanisms, an integrated approach combining 16S rRNA gene sequencing of the gut microbiota and untargeted liver metabolomics was employed. Furthermore, statistical correlation analyses were conducted to explore potential interactions between microbial compositional changes and host metabolic or phenotypic alterations. The main objectives of this study were to assess the efficacy of inactivated YSe as a postbiotic selenium source in alleviating ALD compared to inorganic selenium, to decipher the roles of gut microbiota modulation and hepatic metabolic reprogramming in mediating its hepatoprotective effects, and to establish a scientific basis for the development of YSe-based postbiotic supplements for the prevention of ALD.

## 2. Materials and Methods

### 2.1. Materials and Reagents

The *Saccharomyces cerevisiae* FZU301 strain, originally isolated from traditional *Hongqu* rice wine fermentation systems, was preserved in Yeast Extract Peptone Dextrose (YEPD) broth supplemented with 15% (*v*/*v*) glycerol at −80 °C in a refrigerator. Sodium selenite (Na_2_SeO_3_) was obtained from China National Pharmaceutical Group Co., Ltd. (Shanghai, China). Antioxidant and biochemical assay kits were purchased from Elabscience^®^ Biotech. Co., Ltd. (Wuhan, China). All other chemicals and reagents used were procured from Aladdin Biotech Co., Ltd. (Shanghai, China).

### 2.2. Preparation of Inactivated Se-Enriched Yeast (YSe)

The preparation of selenium-enriched yeast (YSe) was adapted from a previously established protocol [18], with minor modifications. Briefly, the *S. cerevisiae* FZU301 strain was activated in YEPD medium (2% inoculum) under controlled conditions (30 °C for 12 h). Sodium selenite was then aseptically added to achieve a final concentration of 10 μg/mL Se, followed by extended fermentation at 30 °C for 48 h. Control cultures without selenium supplementation (non-selenized yeast, Y) were maintained under identical conditions (37 °C for 48 h). After cultivation, both YSe and Y cells were harvested via centrifugation and freeze-dried through vacuum freeze-dryer, and then stored in a 4 °C refrigerator for further characterization. Prior to animal administration, YSe and Y samples underwent thermal inactivation treatment (100 °C, 30 min) to eliminate viable cells and prevent potential ethanol metabolism by yeast.

### 2.3. Characterization of Se-Enriched Yeast (YSe)

The selenium content in the dried YSe and Y powders was quantified using an Agilent 5800 ICP-OES (Agilent, Santa Clara, CA, USA). The morphological changes of *S. cerevisiae* FZU301 before and after selenium enrichment were analyzed using scanning electron microscopy (SEM, Verios G4 UC, FEI Ltd., Hillsboro, OR, USA) coupled with energy dispersive X-ray spectroscopy (EDX, Quanta FEG 250, FEI Ltd., Hillsboro, OR, USA), following a previously described method [19]. Additionally, the samples were subjected to Fourier-transform infrared (FT-IR) spectroscopy (Vetex70, Bruker, Karlsruhe, Germany) within the range of 400–4000 cm^−1^ to evaluate potential structural changes in cellular biomolecules induced by selenium enrichment.

### 2.4. Animals and Experimental Design

**Figure 1:**
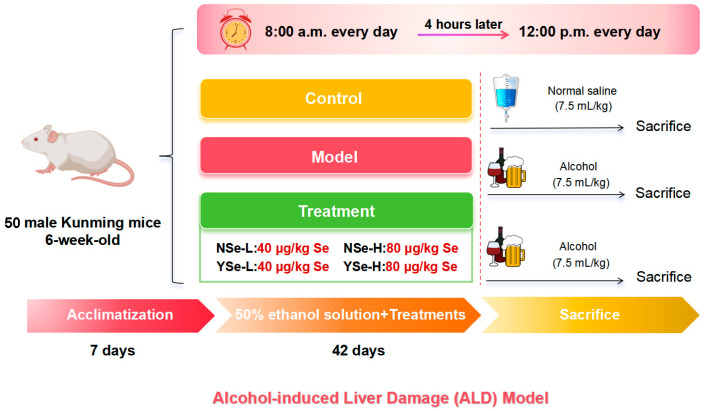
Establishment of ALD model and schematic diagram of drug administration schedule.

This study strictly adhered to the 3R (Replacement, Reduction, Refinement) principles, and all efforts were made to minimize animal suffering. Fifty 6-week-old male pathogen-free Kunming mice (HFK Co., Ltd., Beijing, China) were group-housed in a standard barrier environment. Bedding was changed three times per week, a 12 h light/dark cycle was maintained, and food and water were provided ad libitum. Following a 7-day acclimatization period, all mice were randomly assigned to six groups: Control group (*n* = 8), Model group (*n* = 10), NSe-L group (low-dose sodium selenite, *n* = 8), NSe-H group (high-dose sodium selenite, *n* = 8), YSe-L group (low-dose inactivated YSe, *n* = 8), and YSe-H group (high-dose inactivated YSe, *n* = 8). At 8:00 a.m. daily, the NSe-L and NSe-H groups received oral gavage of sodium selenite at 40 µg/kg and 80 µg/kg body weight, respectively. The YSe-L and YSe-H groups were administered inactivated YSe at equivalent selenium doses. Four hours later, all groups except the Control group were intragastrically administered 50% (*v*/*v*) ethanol at 7.5 mL/kg, while the Control group received an equivalent volume of normal saline. The daily intake of selenium in mice was determined based on human recommended daily intake and interspecies dose conversion principles [12,20]. Following a 42-day intervention, all mice were fasted for 12 h and euthanized by cervical dislocation under deep anesthesia induced by isoflurane. The specific animal experiment process is shown in Figure 1. Blood and tissue samples were collected and processed as described. Serum was isolated by centrifugation (3000 rpm, 25 °C) and stored at −20 °C for biochemical analysis. Organ indices (liver, kidney, spleen) were calculated based on organ weight. Liver and cecal contents were snap-frozen in liquid nitrogen and stored at −80 °C for subsequent analyses. All animal procedures were approved by the Ethics Committee of Institute of Food Science and Technology, Fuzhou University (Approval No.: FZU-IFST-2021018).

### 2.5. Serum and Liver Biochemical Assays

Serum levels of triglycerides (TGs), total cholesterol (TC), low-density lipoprotein cholesterol (LDL-C), high-density lipoprotein cholesterol (HDL-C), aspartate aminotransferase (AST), and alanine aminotransferase (ALT) were measured using a fully automated biochemical analyzer. Liver homogenates were prepared in sterile saline and centrifuged (12,000× *g*, 4 °C, 10 min) to obtain supernatants. Hepatic levels of TC, TG, bile acids (BAs), lactate dehydrogenase (LDH), alcohol dehydrogenase (ADH), glutathione (GSH), superoxide dismutase (SOD), malondialdehyde (MDA), and catalase (CAT) were quantified using commercial assay kits (Jiancheng Bioengineering Institute, Nanjing, China).

### 2.6. Histopathological Analysis of Liver Tissue

Liver tissues were fixed in 4% paraformaldehyde, dehydrated in a graded ethanol series, embedded in paraffin, and sectioned at 3 μm thickness. The sections were deparaffinized, rehydrated, and stained with hematoxylin and eosin (H&E). Histological changes were examined using an optical microscope (Olympus, Tokyo, Japan), and representative images were captured.

### 2.7. Intestinal Microbiota Sequencing Analysis

Genomic DNA was extracted from fecal samples using a commercial DNA extraction kit (Mo Bio Laboratories, Carlsbad, CA, USA). The V3-V4 hypervariable regions of bacterial 16S rRNA genes were amplified using universal primers 341F/806R and subjected to paired-end sequencing on the Illumina MiSeq platform (Majorbio Co., Ltd., Shanghai, China). Sequence quality control was performed using QIIME2 (v2019.7), and bacterial taxonomic profiles were assigned based on the GreenGenes database (v13.8). Multivariate statistical analyses, including principal component analysis (PCA), hierarchical cluster analysis (HCA), and Welch’s *t*-test (STAMP v2.1.3), were conducted to evaluate gut microbiota composition and intergroup differences. Correlation analyses between microbial taxa and biochemical parameters were visualized using the pheatmap package in R (v4.1.2) and Cytoscape (v3.10.1).

### 2.8. Fecal Short-Chain Fatty Acids (SCFAs) Detection

Fecal SCFAs were extracted and quantified following a previously established protocol [11,21]. Briefly, 100 mg lyophilized fecal sample was suspended in 1 mL ultrapure water, vortexed (2500 rpm, 2 min), acidified with 600 μL of 20% (*v*/*v*) sulfuric acid, and mixed with 500 μL n-butanol. The mixture was centrifuged, and the supernatant was collected after three repeated extractions. The supernatant was filtered through a 0.22 μm membrane and analyzed using gas chromatography (Agilent 7890B, CA, USA). A standard curve was constructed for quantitative analysis of SCFAs concentrations.

### 2.9. Liver Metabolome Assay

Liver tissue samples (50 mg) were homogenized in 1 mL extraction solvent (acetonitrile: methanol: water = 2:2:1, *v*/*v*/*v*) using ultrasonic disruption at 4 °C for 10 min. The homogenates were centrifuged (12,000× *g*, 4 °C, 10 min), and the supernatants were vacuum-dried and reconstituted in 200 μL of 80% methanol. After filtration through 0.22 μm membranes, the extracts were analyzed using an Agilent 1290 Infinity UPLC system coupled with a 6530 QTOF-MS/MS mass spectrometer. Quality control (QC) samples were prepared by pooling equal volumes of all experimental samples. Raw data were processed using Mass Profiler Professional 14.5, and multivariate statistical methods, including PCA, PLS-DA, and OPLS-DA, were applied using MetaboAnalyst 5.0 to identify significant metabolic alterations

### 2.10. Statistical Analysis

All data were analyzed using GraphPad Prism 9.5 and SPSS 23.0. Intergroup differences were assessed by one-way ANOVA, followed by Tukey’s Honest Significant Difference (HSD) post hoc test for multiple comparisons. Differential metabolites between groups were selected based on a variable importance in projection (VIP) score from the OPLS-DA model of >1.0 and a *p*-value from Student’s *t*-test of < 0.05. Pathway analysis of liver metabolites with significant difference was performed on MetaboAnalyst 5.0. Data are presented as mean ± standard deviation (SD). * *p* < 0.05 and ** *p* < 0.01 versus Control group; ^#^ *p* < 0.05 and ^##^ *p* < 0.01 versus Model group.

## 3. Results and Discussion

### 3.1. Morphological and Structural Characterization of YSe

**Figure 2:**
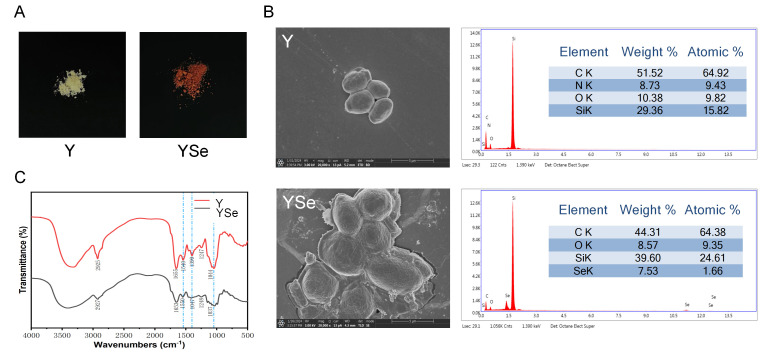
Characterization of the external morphology, micromorphology and chemical functional group structure of Y and YSe. (**A**) The external morphology of Y and YSe; (**B**) The micromorphology of Y and YSe observed by SEM and EDX spectra; (**C**) The chemical functional group structure of Y and YSe revealed by FTIR analysis.

One of the most prominent macroscopic indicators of successful selenium enrichment in yeast is the change in cell color, typically associated with the excitation of surface plasma vibrations of elemental selenium [22]. As shown in Figure 2A, non-enriched yeast cells appeared white, whereas YSe exhibited a distinct red coloration. This transformation is attributed to the yeast’s capacity to biotransform inorganic selenium into red, monomeric elemental selenium and subsequently secrete it as nanoparticles outside the cell wall [23]. Morphological analysis via SEM revealed notable differences between non-enriched yeast (Y) and YSe. As seen in Figure 2B, Y cells displayed a smooth, uniform surface devoid of any particulate matter. In contrast, YSe cells appeared larger, rougher, and were covered with small granular structures, likely corresponding to selenium nanoparticles formed during the enrichment process. To confirm the presence of elemental selenium on the yeast surface, energy-dispersive X-ray spectroscopy (EDX) was employed. Compared to the control sample (Y), the YSe sample exhibited a distinct energy peak at approximately 1.4 keV, indicative of a high concentration of zero-valent selenium on the surface, supporting the formation of selenium nanoparticles [24].

FT-IR analysis further confirmed the successful incorporation of selenium into the yeast matrix (Figure 2C). The most notable change was the obvious weakening of the broad absorption band at 3720–3000 cm^−1^ in YSe, which corresponds to the stretching vibrations of -NH_2_ and -OH groups. This suggests potential chemical interactions between selenium and these functional groups on yeast proteins and polysaccharides. Additionally, the appearance of a new absorption peak at 1540 cm^−1^ in YSe, characteristic of amide II bonds (N-H bending and C-N stretching), provides direct evidence for the involvement of yeast proteins in selenium binding [25]. The presence of peaks associated with -C-O- and -COOH vibrations further supports the interaction between selenium and carbohydrate components of the cell wall [26]. Collectively, these morphological and spectral analyses provide compelling evidence that yeast successfully assimilates and transforms inorganic selenium into elemental selenium nanoparticles. These structural modifications likely enhance the bioavailability and functional properties of YSe, laying the foundation for its potential biological effects.

### 3.2. Effect of Inactivated YSe on Body Weight and Organ Indexes

**Figure 3:**
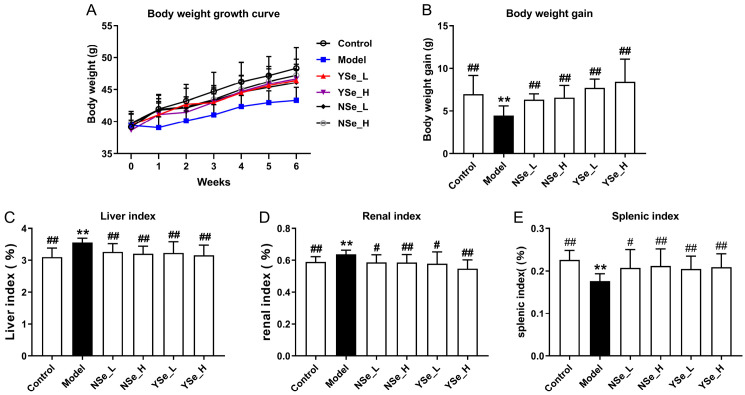
Effects oral administration of inactivated YSe and inorganic Se on growth performance and organ indices in alcohol-exposed mice. (**A**) Body weight growth curves; (**B**) Body weight gain; (**C**) Liver index; (**D**) kidney index; (**E**) spleen indexes. Sample sizes: model group, *n* = 10; other groups, *n* = 8. Statistical significance was determined by one-way ANOVA followed by Tukey’s post hoc test. ** *p* < 0.01 versus Control group; ^#^ *p* < 0.05 and ^##^ *p* < 0.01 versus Model group.

The effects of inactivated YSe on the growth performance and organ indices of alcohol-treated mice were summarized in Figure 3. Notably, mice in the Model group (alcohol-treated, untreated with selenium) exhibited significantly lower body weights compared to the Control group throughout the 6-week experimental period (Figure 3A,B), indicating that chronic alcohol consumption impairs overall growth and body weight gain. This observation aligns with previous findings that chronic alcohol consumption may impair growth and nutrient metabolism, reflecting systemic physiological stress [27]. In addition, the liver and kidney indices (organ weight relative to body weight) were significantly elevated in the Model group compared to the Control group (*p* < 0.01), while the spleen index was markedly reduced (*p* < 0.01) (Figure 3C–E). These alterations suggest that alcohol-induced toxicity leads to hepatomegaly and nephromegaly, possibly due to fat accumulation and inflammation, while spleen atrophy may reflect systemic immune dysfunction. In contrast, oral administration of both sodium selenite (NSe) and inactivated YSe significantly improved body weight and spleen index in alcohol-treated mice, while reducing liver and kidney indices (*p* < 0.05). These findings indicate that both selenium forms exert protective effects, but inactivated YSe demonstrated superior efficacy compared to NSe in several parameters, potentially due to its enhanced bioavailability and antioxidant capacity. These findings underscore the multi-targeted hepatoprotective and systemic regulatory effects of inactivated YSe in the context of alcohol-induced organ dysfunction.

### 3.3. Effect of Inactivated YSe on Serum Biochemical Phenotypes

**Figure 4:**
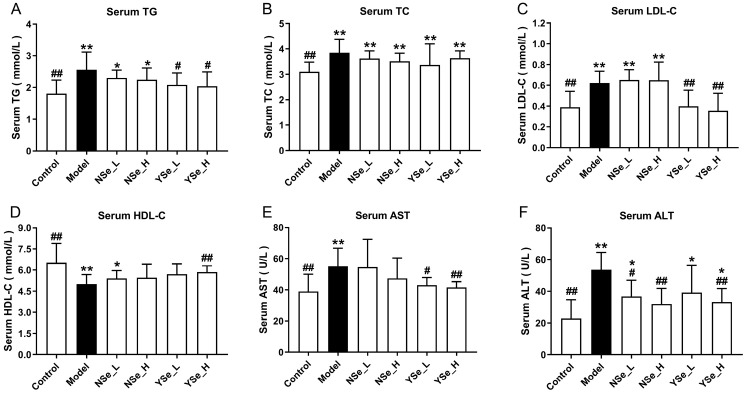
Effects of inactivated YSe and inorganic Se on serum biochemical phenotypes in over-drinking mice. (**A**) Serum TC; (**B**) Serum TG; (**C**) Serum LDL-C; (**D**) Serum HDL-C; (**E**) Serum AST; (**F**) Serum ALT. Sample sizes: model group, *n* = 10; other groups, *n* = 8. Statistical significance was determined by one-way ANOVA followed by Tukey’s post hoc test. * *p* < 0.05 and ** *p* < 0.01 versus Control group; ^#^ *p* < 0.05 and ^##^ *p* < 0.01 versus Model group.

At the 6th week of alcohol exposure, serum biochemical parameters (including TG, TC, LDL-C and HDL-C) were assessed in mice (Figure 4). Compared to the Control group, excessive alcohol consumption significantly elevated serum levels of TGs, TC, and LDL-C (*p* < 0.01), while markedly suppressing HDL-C levels (decreased by 23.34%). It is well established that elevated LDL-C and TG levels are closely associated with lipid metabolism disorders, such as hyperlipidemia and hyperglycemia [18]. Conversely, HDL-C plays a protective role in lipid metabolism by facilitating the reverse transport of cholesterol from peripheral tissues to the liver, thereby lowering plasma TC levels and reducing the risk of atherosclerosis [28]. Moreover, serum AST and ALT activities were significantly elevated in the Model group compared to the Control group (increased by 41.60% and 134.64%, respectively), indicating substantial hepatic injury, as these enzymes are commonly used as clinical markers of liver damage. NSe administration only significantly reduced ALT levels in alcohol-exposed mice (*p* < 0.05), but showed no significant effects on TGs, TC, and LDL-C (*p* > 0.05). In contrast, even low-dose inactivated YSe significantly lowered TG, LDL-C, and AST levels simultaneously (*p* < 0.05). High-dose YSe intervention increased HDL-C by 16.83% compared to the Model group, while reducing ALT and AST levels by 38.69% and 24.67%, respectively. These findings collectively suggest that inactivated YSe exerts a more potent hepatoprotective effect than NSe, particularly in regulating lipid homeostasis and attenuating alcohol-induced liver injury. The therapeutic outcomes observed here corroborate the established superiority of organic selenium compounds (selenium-enriched peptides) over their inorganic counterparts (sodium selenite), as previously reported by Li et al. in the context of alcohol-induced liver injury [29].

### 3.4. Effects of Inactivated YSe on Liver Biochemical Parameters

**Figure 5:**
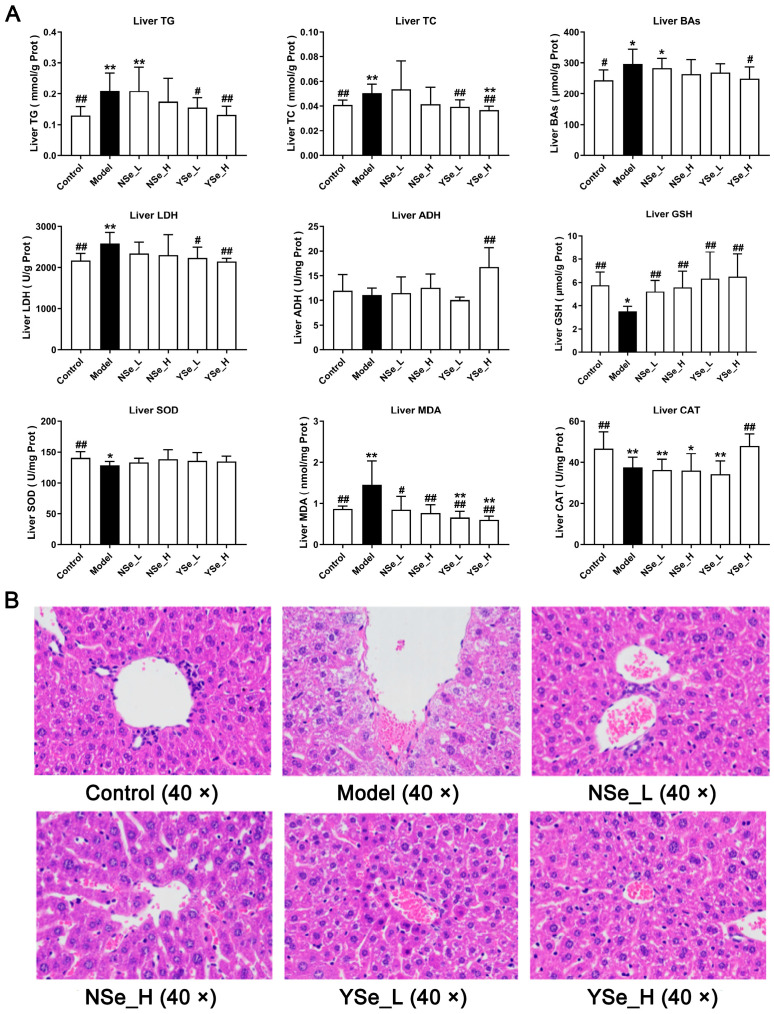
Effects of inactivated YSe and inorganic Se intervention on liver biochemical phenotypes and liver histopathological features in over-drinking mice. (**A**) Biochemical parameters; (**B**) Histopathological feature. Sample sizes: model group, *n* = 10; other groups, *n* = 8. Statistical significance was determined by one-way ANOVA followed by Tukey’s post hoc test. * *p* < 0.05 and ** *p* < 0.01 versus Control group; ^#^ *p* < 0.05 and ^##^ *p* < 0.01 versus Model group.

Alcohol exposure significantly increased hepatic TG, TC and bile acids (BAs) levels compared to the Control group (*p* < 0.01) (Figure 5A), indicating exacerbated liver damage. Excess TC in the liver is often converted into LDL-C, contributing to elevated circulating LDL-C levels and accelerating the progression of lipid metabolism-related diseases [30]. Importantly, high-dose inactivated YSe treatment significantly reduced hepatic TG, TC and BAs levels (*p* < 0.05), whereas NSe treatment showed no significant effect on these parameters (Figure 5A). Lactate dehydrogenase (LDH) serves as a sensitive indicator of liver injury and was markedly elevated in the Model group compared to the Control group (*p* < 0.01). Alcohol metabolism primarily involves hepatic oxidation mediated by alcohol dehydrogenase (ADH) and aldehyde dehydrogenase (ALDH) [27]. High-dose inactivated YSe administration significantly decreased hepatic LDH activity while increasing ADH activity in ALD mice (*p* < 0.01), indicating improved alcohol metabolism and reduced cellular damage.

Oxidative stress is a well-documented contributor to ALD progression in both mice and humans [31]. Chronic alcohol intake significantly reduced hepatic levels of glutathione (GSH), superoxide dismutase (SOD), and catalase (CAT) (*p* < 0.01), while significantly increasing malondialdehyde (MDA) levels (*p* < 0.01) (Figure 5A). MDA, a byproduct of lipid peroxidation, is a widely accepted biomarker of oxidative damage [32]. GSH, SOD, and CAT are key components of the endogenous antioxidant defense system, essential for maintaining redox homeostasis and protecting cells from oxidative stress [33]. Intervention results showed that both NSe and YSe significantly restored hepatic reduced GSH levels in alcohol-exposed mice (*p* < 0.01). As the obligate substrate of the selenium-dependent enzyme GSH-Px, GSH abundance directly dictates GSH-Px activity [34]. The organic selenium (selenomethionine) in YSe can be randomly incorporated into liver proteins during synthesis, forming a “selenium reservoir” that continuously supplies precursors for GSH and GSH-Px regeneration [35]. Consequently, the GSH increment in the YSe group was 16.33% higher than in the NSe group. Moreover, only high-dose YSe significantly elevated CAT activity (*p* < 0.01), while no treatment had a significant effect on SOD activity.

In summary, by optimizing the selenium metabolism process, YSe more efficiently supports the synthesis and function of hepatic selenoproteins, thereby providing a more comprehensive antioxidant protection than traditional inorganic selenium and offering a superior intervention strategy for alcohol-induced liver injury.

### 3.5. Effects of Inactivated YSe on Liver Histopathological Features

Histopathological changes in liver tissues were assessed using hematoxylin and eosin (H&E) staining (Figure 5B). Mice in the alcohol-exposed groups exhibited characteristic features of ALD, including widespread microvesicular steatosis, extensive hepatocyte swelling, small extramedullary hematopoietic lesions, and pronounced inflammatory cell infiltration [10]. These alterations are indicative of the progressive liver damage associated with chronic alcohol consumption. However, both NSe and inactivated YSe interventions demonstrated certain degrees of histological improvement. Notably, inactivated YSe-treated mice exhibited reduced cellular border blurring, diminished inflammatory cell infiltration, and improved overall hepatocyte morphology compared to the Model group. These histological observations corroborate the biochemical findings and further support the conclusion that inactivated YSe is effective in mitigating ALD. Moreover, inactivated YSe appears to confer greater hepatoprotective benefits than NSe, as evidenced by both structural and functional improvements in liver tissue.

### 3.6. Effects of Inactivated YSe on Fecal SCFAs Levels

**Figure 6:**
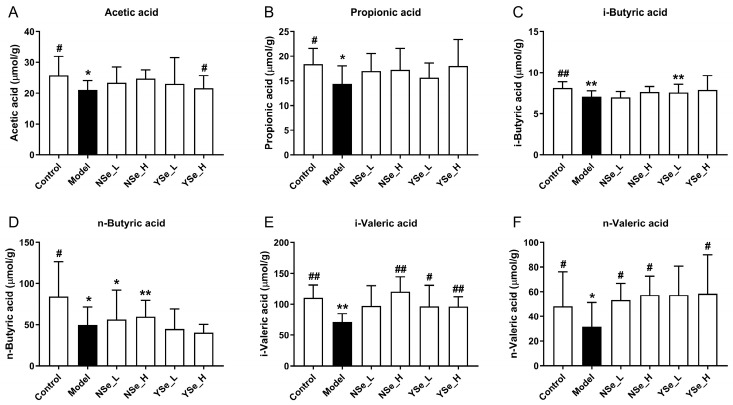
Effects of inactivated YSe and inorganic Se intervention on the levels of fecal SCFAs in over-drinking mice. (**A**) Acetic acid; (**B**) Propionic acid; (**C**) i-Butyric acid; (**D**) n-Butyric acid; (**E**) i-Valeric acid; (**F**) n-Valeric acid. Sample sizes: model group, *n* = 10; other groups, *n* = 8. Statistical significance was determined by one-way ANOVA followed by Tukey’s post hoc test. * *p* < 0.05 and ** *p* < 0.01 versus Control group; ^#^ *p* < 0.05 and ^##^ *p* < 0.01 versus Model group.

A diverse range of dietary components, including probiotics, prebiotics, and postbiotics, are known to confer health benefits by modulating the composition and function of the gut microbiota [36]. A key mechanism underpinning these benefits is the microbial production of SCFAs, which are crucial metabolites that play pivotal roles in maintaining host energy metabolism and promoting intestinal and systemic health [5]. In this study, the main SCFAs levels of the control group mice were significantly higher than those of the alcohol-exposed mice (*p* < 0.05) (Figure 6). Acetic acid has been shown to improve hepatic lipid metabolism by modulating taurine-conjugated bile acid metabolism [37]. Propionic acid functions as an energy source and inhibits hepatic cholesterol synthesis [38]. Butyric acid supports intestinal mucosal cell growth and repair and reduces DNA damage in epithelial cells [39]. Notably, inactivated YSe intervention significantly elevated the levels of acetate, i-butyric, i-valeric, and n-valeric acids in the stools of alcohol-exposed mice (*p* < 0.05). In comparison, NSe treatment increased the levels of n-butyric, i-valeric and n-valeric acids to a lesser extent. Overall, these findings indicate that inactivated YSe effectively counteracts alcohol-induced depletion of SCFAs, thereby supporting metabolic homeostasis.

### 3.7. Effects of Inactivated YSe on Intestinal Microflora

Emerging evidence highlights the critical role of gut microbiota in the pathogenesis of liver diseases, including alcoholic liver disease (ALD) [40]. In this study, high-throughput 16S rRNA sequencing was employed to assess the impact of YSe and NSe interventions on the gut microbial composition of mice subjected to chronic alcohol exposure. Intestinal microflora alpha diversity analysis revealed that although alcohol exposure did not cause statistically significant reductions in microbial species richness or diversity in the Model group, overall levels showed a decreasing trend. Notably, compared with the Model group, YSe_H intervention significantly reversed the changing trends in both the Shannon and Simpson indices, demonstrating its definite ameliorative effect on the structure of the gut microbiota (Figure S1). Principal coordinates analysis (PCoA) revealed distinct clustering patterns between the Control and Model groups, indicating that alcohol exposure significantly alters intestinal microbiota composition in mice (Figure S2A,B). Notably, both high-dose NSe and YSe treatments partially restored the microbial dysbiosis induced by chronic alcohol intake, as confirmed by hierarchical clustering analysis (Figure S2C).

**Figure 7:**
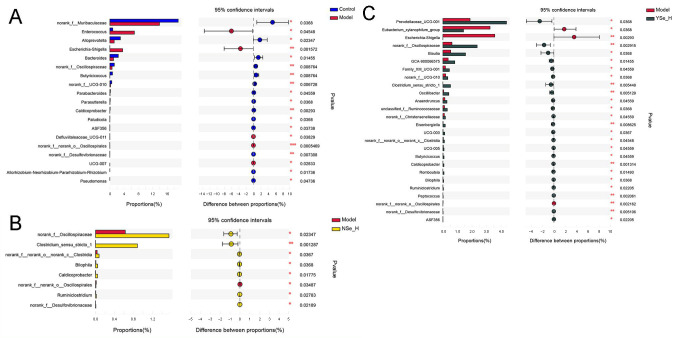
The differences in the abundance of intestinal bacterial genera between different experimental groups. (**A**) Control group versus Model group; (**B**) NSe_H group versus Model group; (**C**) YSe_H group versus Model group. Sample sizes: model group, *n* = 10; other groups, *n* = 8. For comparisons between groups, statistical significance was determined by the Wilcoxon rank-sum test (* *p* < 0.05, ** *p* < 0.01).

At the genus level, Wilcoxon rank sum test identified significant differences in the relative abundances of 19 key microbial taxa between the Model and Control groups (Figure 7). Specifically, the abundances of opportunistic pathogens such as *Enterococcus* and *Escherichia-Shigella* were significantly increased in the Model group. In contrast, the relative abundances of the beneficial bacterial genera, including norank_f_*Muribaculaceae*, *Alloprevotella*, *Bacteroides*, norank_f_*Oscillospiraceae*, *Butyricicoccus*, norank_f_UCG-010, *Parabacteroides*, *Parasutterella*, and *Caldicoprobacter*, were significantly reduced. *Enterococcus* is a conditional pathogen commonly found in the gastrointestinal tract, has been associated with various infectious diseases, including endocarditis, urinary tract infections, and liver injury [41]. Increased abundance of *Escherichia–Shigella* has also been reported in alcohol-fed mice, indicating a potential link between gut dysbiosis and systemic inflammation [42]. Conversely, SCFAs-producing bacterial genera such as *Muribaculaceae*, *Alloprevotella*, *Oscillospiraceae*, *Butyricicoccus*, and *Parabacteroides* have been shown to support intestinal barrier function and immune homeostasis [43].

High-dose NSe intervention significantly increased the relative abundances of norank_f_*Oscillospiraceae*, norank_c_*Clostridia*, *Bilophlia*, *Caldicoprobacter*, *Ruminiclostridium*, norank_f_*Desulfovibrionaceae*, *Clostridium*_sensu_stricto_1, while decreasing norank_f_*Oscillospirales*. Among these, *Clostridium*_sensu_stricto_1 is a key anaerobic fermentative bacterium capable of metabolizing carbohydrates and amino acids, contributing to intestinal homeostasis by suppressing pathogenic bacteria such as *Escherichia coli* [44,45]. *Desulfovibrionaceae* is capable to reduce environmental sulfate levels, and produce acetate by lactate and hydrogen metabolism [46]. Interestingly, high-dose of inactivated YSe treatment significantly elevated the relative abundances of *Prevotellaceae*_UCG-001, norank_f_*Oscillospiraceae*, *Blautia*, GCA-900066575, norank_f_UCG-010, *Peptococcus*, *Bilophila*, *Oscillibacter*, *Clostridium*_sensu_stricto_1, *Butyricicoccus*, *Anaerotruncus*, Family_X UCG-001,UCG-005, unclassified_f_*Ruminococcaceae*, *Eisenbrngirlla*, UCG-003, norank_f_*Christensenellaceae*, norank_c_*Clostridia*, *Caidicoprobacter*, *Romboutsia*, norank_f_*Desulfoviibrionaceae*, *Ruminiclostridium*, while reducing *Eubacterium_xylanophium*_group, *Escherichia-Shigella*, norank_o_*Oscilliumospirates*. Notably, *Prevotellaceae*, a family within the phylum *Bacteroidetes*, is known to modulate inflammatory responses and maintain mucosal immunity [47]. *Blautia* can up-regulate the expression of tight junction proteins. These proteins act as the “cement” between intestinal epithelial cells, strengthening the physical barrier of the intestine and preventing harmful substances and bacteria in the intestine from entering the bloodstream, thereby avoiding systemic inflammation [32]. *Oscillibacter*, *Anaerotruncus*, *Ruminococcaceae*, *Butyricicoccus*, and *Ruminiclostridium* are all important SCFAs producers that mediate crosstalk between the gut microbiota and the immune system, influencing both inflammatory and metabolic disorders [48]. Among these, clinical-metagenomic studies have revealed that as NAFLD fibrosis progresses, the abundance of *Ruminococcaceae* decreases, while serum bile acid synthesis increases. Supplementation with corresponding bacterial strains in animal models has been shown to reverse this phenomenoue [49]. Additionally, butyrate produced by *Oscillibacter* and *Ruminococcaceae* inhibits histone deacetylase, promotes the differentiation of colonic Treg cells, upregulates IL-10, and downregulates pro-inflammatory cytokines such as TNF-α, IL-6, and MCP-1, thereby reducing systemic low-grade inflammation and indirectly protecting the liver [50]. Furthermore, the *Eubacterium_xylanophilum*_group has been reported to improve lipid metabolism by suppressing hepatic triglyceride accumulation [51]. Collectively, then, suggesting their hepatoprotective role via bile acid–liver disease findings suggest that both NSe and inactivated YSe interventions can modulate the gut microbiota composition in alcohol-exposed mice, promoting a healthier microbial balance and potentially contributing to the alleviation of ALD.

### 3.8. Correlation Between Key Intestinal Bacteria with Biochemical Parameters

**Figure 8:**
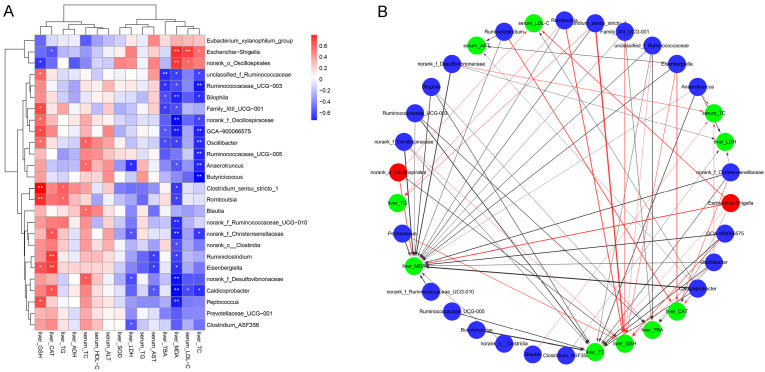
Correlation between intestinal bacteria and biochemical phenotypes. (**A**) Correlation heat map. Red and blue colors represent positive and negative correlations, respectively. Statistical significance was denoted as * *p* < 0.05 and ** *p* < 0.01. (**B**) Correlation network. Red nodes: intestinal bacteria decreased by inactivated YSe intervention; blue nodes: intestinal bacteria increased by inactivated YSe intervention; green nodes: biochemical parameters. Solid red and the black lines represent positive and negative correlations, respectively. Line width represents the strength of correlation. Only significant correlations (|r| > 0.6, *p* < 0.05) were plotted in the network.

To investigate the potential associations between gut microbiota modulation by inactivated YSe and liver health outcomes, Spearman correlation analysis was conducted between the relative abundances of key intestinal bacteria and serum/hepatic biochemical parameters. As illustrated in Figure 8, the relative abundances of *Eubacterium xyanophilum* group, *Escherichia-Shigella*, and norank_o_*Oscillospirales* were positively correlated with serum LDL-C, hepatic TC, MDA and TBA levels, while showing negative correlations with serum ALT, HDL-C and TC levels, as well as hepatic SOD, ADH, CAT, GSH, and TG levels. In contrast, several beneficial taxa, including *Ruminococcaceae* UCG-003, *Bilophila*, Family_XIII UCG-001, GCA-900066575, *Oscillibacter*, *Ruminococcaceae* UCG-005, *Anaerotruncus*, *Butyricoccus*, *costridium*_sensu_stricto_1, norank_c_*Clostridia*, *Romboutsia*, norank_f_*Ruminococcaceae* UCG-010, *Ruminiclostridium Eisenbergiella*, *Caldicoprobacter*, *Peptococcus*, *Prevotellaceae*_UCG-001, *Clostridium*_ASF356 were inversely correlated with serum LDL-C, and hepatic TC, MDA and TBA levels, but positively correlated with serum ALT, HDL-C, TC, and hepatic SOD, ADH, CAT, GSH, and TG levels. These results indicate that these beneficial bacteria may play a protective role in ALD by enhancing antioxidant defenses and lipid metabolism. These results indicate that these beneficial bacteria may play a protective role in ALD by enhancing antioxidant defenses and lipid metabolism.

### 3.9. Effects of Inactivated YSe on Liver Metabolome

**Figure 9:**
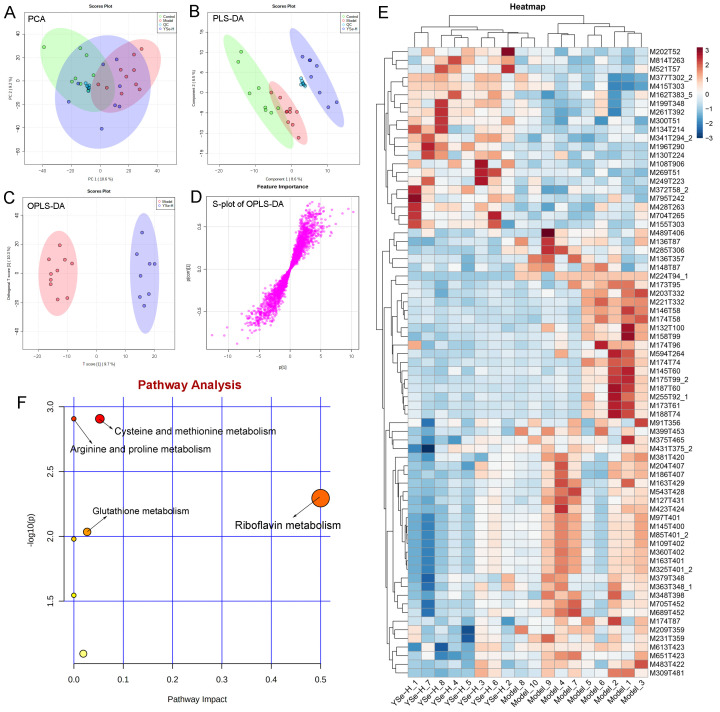
Comparative liver metabolomics analysis in positive ion mode. (**A**) PCA scoring plot; (**B**) PLS-DA scoring plot; (**C**) OPLS-DA scoring plot; (**D**) S-loading plot; (**E**) Differentially abundant metabolites between the model and YSe_H groups (VIP > 1.0, *p* < 0.05) were displayed in the heatmap, with red and blue representing increased and decreased abundance, respectively; (**F**) Pathway enrichment of liver differential metabolites. Sample sizes: model group, *n* = 10; other groups, *n* = 8.

**Figure 10:**
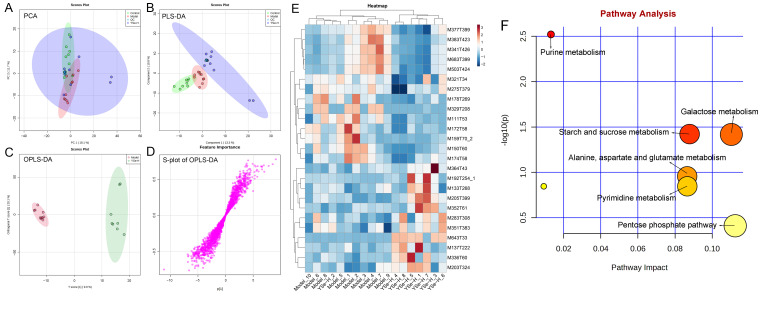
Comparative liver metabolomics analysis in negative ion mode. (**A**) PCA scoring plot; (**B**) PLS-DA scoring plot; (**C**) OPLS-DA scoring plot; (**D**) S-loading plot; (**E**) Differentially abundant metabolites between the model and YSe_H groups (VIP > 1.0, *p* < 0.05) were displayed in the heatmap, with red and blue representing increased and decreased abundance, respectively; (**F**) Pathway enrichment of liver differential metabolites. Sample sizes: model group, *n* = 10; other groups, *n* = 8.

To investigate the impact of YSe on hepatic metabolic profiles in alcohol-exposed mice, untargeted metabolomics analysis was conducted using UPLC-QTOF-MS. Principal component analysis (PCA) revealed distinct metabolic profiles between the Control, Model, and YSe groups, with inactivated YSe intervention partially reversing the metabolic disturbances induced by alcohol (Figure 9A and Figure 10A). These findings were corroborated by PLS-DA (Figure 9B and Figure 10B), and OPLS-DA (Figure 9C and Figure 10C), which demonstrated that YSe significantly shifted the metabolic profiles in alcohol-consuming mice, suggesting a potential therapeutic role in ALD. Subsequently, differential metabolites between the Model and YSe-H groups were identified (VIP > 1, *p* < 0.05) (Figure 9D and Figure 10D).

In positive ion mode (ESI+), a total of 73 differential metabolites were identified, with 21 showing increased levels in the YSe-H group compared to the Model group. Notably, metabolites such as n-methylephedrine [M162T383_5], (+)-isomenthone [M155T303], 1-stearoyl-rac-glycerol [M341T294_2], n-palmitoyl-D-sphingosine [M521T57], palmitoyl sphingomyelin [M704T265], cytidine 5′-diphosphocholine [M489T406] were significantly increased in the YSe-H group (Figure 9E and Table S1). Many of these metabolites are involved in antioxidant defense, lipid metabolism, and cell signaling, energy homeostasis, and their depletion may contribute to ALD pathogenesis. For instance, in the lipopolysaccharide (LPS)/D-galactosamine–induced acute liver failure model, ephedrine-type alkaloids (including N-methylephedrine [M162T383_5]) exhibited significant hepatoprotective effects [52]. While 1-stearoyl-rac-glycerol [M341T294_2] promotes hepatic lipolysis and reduces lipid accumulation [53]. (+)-isomenthone [M155T303] inhibits tumor cell growth via TRPM8 pathway regulation [54]. Stearoylcarnitine [M428T263] is crucial for fatty acid oxidation and energy production [55]. Kobusone [M261T392] and isovitexin (IVT) [M415T303] are known for their antioxidant and anti-inflammatory properties [56,57]. Riboflavin [M377T302_2] and 12,13-cis-retinol [M269T51] are essential vitamins involved in metabolic and redox functions [58,59]. Parthenolide [M249T223] belongs to the derivatives of germacranolides that possesses a series of physiological effects, including anti-inflammatory and antioxidant properties, which are beneficial for improving the liver function [39]. In contrast, metabolites such as 2-(5-oxovaleryl) phosphatidylcholine [M594T264] and 1-naphthol [M145T60] was found to induce organ injury through the activation of NLRP3 inflammasomes [60], suggesting that its downregulation by YSe may contribute to liver protection. In negative ion mode (ESI-), 25 differential metabolites were identified, with 11 upregulated in the YSe-H group, including malate [M133T268], 3,4-dihydroxybenzaldehyde [M137T222], D-tryptophan [M203T324], xanthosine [M283T308], and linoleoylglycine [M336T60] (Figure 10E and Table S2). Among them, malate [M133T268], a key intermediate in the tricarboxylic acid (TCA) cycle, has been shown to protect against ethanol-induced hepatocyte damage [61], while 3,4-dihydroxybenzaldehyde [M137T222] (DHB) exhibits potent antioxidant and antimicrobial effects [62]. Metabolites such as 3′,5′-cyclic inosine monophosphate [M329T208] (cIMP), guanine [M150T60], xanthosine [M283T308], thymidine 5′-monophosphate [M643T33] are involved in purine metabolism, which is closely linked to liver injury and metabolic dysfunction [63]. On the other hand, succinic acid n, n-dimethylhydrazide [M159T70_2] may exacerbate liver damage by promoting extracellular matrix protein synthesis [64], and its downregulation following inactivated YSe treatment suggests a role in attenuating fibrotic processes.

KEGG pathway analysis revealed that inactivated YSe-modulated metabolites were enriched in the following pathways including cysteine and methionine metabolism, arginine and proline metabolism, glutathione metabolism, and riboflavin metabolism (Figure 9F), as well as purine metabolism, starch and sucrose metabolism, galactose metabolism, pyrimidine metabolism, alanine, aspartate, and glutamate metabolism, and pentose phosphate pathway (Figure 10F). Complete pathway enrichment results, including adjusted *p*-values and impact scores, are provided in Supplementary Tables S3 and S4. These pathways do not operate in isolation but rather form a coordinated regulatory metabolic network. Specifically, intermediates of purine metabolism serve as precursors for amino acid metabolism, influencing the balance of hepatic amino acids [65]. Furthermore, this finding resonates with previous research demonstrating that yeast extract protects the alcohol-induced liver damage by regulating purine metabolites such as adenosine [66]. Glutathione metabolism acts as a central hub, and its synthesis relies on precursors supplied by amino acid metabolism. The enhancement of this pathway following YSe intervention aligns closely with the marked alleviation of hepatic oxidative stress (Figure 5A). Meanwhile, the pentose phosphate pathway supports cellular antioxidant defense by supplying NADPH, which is essential for maintaining reduced glutathione in its active form, thereby acting in synergy with glutathione metabolism [67]. These findings suggest that YSe exert its effects by systemically reprogramming the hepatic metabolic network to synergistically enhance the organism’s antioxidant response.

### 3.10. Correlation Between Key Intestinal Bacteria with Liver Metabolites

To elucidate the mechanistic basis of YSe action, we integrated data on hepatic metabolic changes with YSe-induced remodeling of the gut microbiota (Figures S3 and S4). Notably, YSe supplementation enriched several beneficial bacterial taxa including *Oscillospiraceae*, *Butyricicoccus*, *Oscillibacter*, *Ruminiclostridium* and *Anaerotruncus*, all of which are established producers of SCFAs. These microbes appeared to coordinately regulate amino acid metabolism and activate hepatoprotective pathways. SCFAs not only contribute to intestinal barrier integrity but also transit via the portal vein to the liver, where they serve as carbon skeletons and metabolic precursors for amino acid and glutathione biosynthesis, thereby systemically enhancing hepatic anabolic and antioxidant capacity [68,69]. In support of this, correlation analyses revealed that these SCFA-producing bacteria were positively associated with key metabolites involved in glutathione and riboflavin metabolism, such as 1-stearoyl-rac-glycerol [M341T294_2] and (–)-riboflavin [M377T302_2] (Figure S3).

In parallel, we observed significant correlations between YSe-modulated bacteria and hepatic metabolites related to purine and amino acid metabolism. Specifically, the conditional pathogen *Escherichia–Shigella*, which was markedly suppressed by YSe, exhibited a negative correlation with hepatic purine metabolites, including thymidine 5′-monophosphate [M643T33] and xanthosine [M283T308] (Figure S4). This finding suggests that overgrowth of *Escherichia–Shigella* may disrupt hepatic purine homeostasis by promoting gut inflammation, compromising intestinal barrier function, and facilitating endotoxin translocation to the liver [70,71]. Thus, YSe-mediated suppression of this pathobiont likely contributes to the restoration of normal purine metabolism in the liver.

Collectively, these findings provide direct evidence at the “microbiota–metabolite” interaction level that YSe modulates host liver metabolism via gut microbial remodeling, elucidating a key pathway through which it mediates protection against alcohol-induced liver injury.

## 4. Conclusions

This study systematically elucidated for the first time the protective effect and mechanism of inactivated selenium-enriched yeast (YSe) as a novel postbiotic preparation in alleviating alcoholic liver injury (ALD). The results show that inactivated YSe can significantly improve lipid accumulation and oxidative stress in the liver, thereby effectively delaying the progression of the disease. The integration of 16S rRNA sequencing and metabolomics analysis further revealed that inactivated YSe has a dual regulatory function. It can reshape the structure of the intestinal flora, promote the proliferation of beneficial bacteria and inhibit the growth of potential pathogenic bacteria. It simultaneously regulates the liver metabolic profile, influencing multiple key pathways such as glutathione metabolism, purine metabolism, and riboflavin metabolism, jointly maintaining liver metabolic homeostasis. These findings suggest that inactivated YSe exerts a liver-protective effect through a multi-target synergistic mechanism along the gut-liver axis. This study provides a new perspective for understanding the potential of postbiotics in the intervention of metabolic liver diseases and also lays a theoretical foundation for the development of safe and efficient selenium-enriched functional foods and nutritional preparations. Further clinical research is still needed to verify its effectiveness in the human body and to further explain its mechanism of action.

## Data Availability

The original contributions presented in the study are included in the article, further inquiries can be directed to the corresponding author.

## Supplementary Materials

The following supporting information can be downloaded at: https://www.mdpi.com/article/10.3390/foods14244209/s1, Figure S1: Effects of inactivated YSe and inorganic Se interventions on the alpha diversity of intestinal flora in ALD mice. (A) ACE index. (B) Chao index. (C) Shannon index. (D) Simpson index. Sample sizes: model group, *n* = 10; other groups, *n* = 8. Statistical significance was determined by one-way ANOVA followed by Tukey’s post-hoc test. ^#^ *p* < 0.05 and ^##^ *p* < 0.01 versus Model group. Figure S2: Effects of inactivated YSe and inorganic Se intervention on the composition of intestinal flora in ALD mice at the genus level. (A, B) Principal coordinate analysis (PCoA). (C) Hierarchical cluster analysis diagram. Sample sizes: model group, *n* = 10; other groups, *n* = 8. Figure S3: Spearman’s Correlation analysis between key intestinal microbes and differential metabolites in the ESI+ mode. Red and blue colors represent positive and negative correlations, respectively. Statistical significance was denoted as * *p* < 0.05 and ** *p* < 0.01. Figure S4: Spearman’s Correlation analysis between key intestinal microbes and differential metabolites in the ESI- mode. Red and blue colors represent positive and negative correlations, respectively. Statistical significance was denoted as * *p* < 0.05 and ** *p* < 0.01. Table S1: The abundance of differential metabolites between the Model and YSe-H groups by UPLC-QTOF/MS in ESI+ mode. Differential metabolites between groups were selected based on VIP > 1.0 and *p* < 0.05. Table S2: The abundance of differential metabolites between the Model and YSe-H groups by UPLC-QTOF/MS in ESI− mode. Differential metabolites between groups were selected based on VIP > 1.0 and *p* < 0.05. Table S3: Result from pathway analysis of liver metabolites in ESI+ mode. The Total Cmpd is the total number of compounds in the pathway; the −log10 (p) is obtained by taking the negative logarithm (base 10) of the raw *p*-value; the FDR p is the *p* value adjusted using False Discovery Rate; the Impact is the pathway impact value calculated from pathway topology analysis. Table S4: Result from pathway analysis of liver metabolites in ESI- mode. The Total Cmpd is the total number of compounds in the pathway; the −log10 (p) is obtained by taking the negative logarithm (base 10) of the raw *p*-value; the FDR p is the *p* value adjusted using False Discovery Rate; the Impact is the pathway impact value calculated from pathway topology analysis.
