# Supplementary material for: Gut Microbiota and Liver Metabolism Regulation Mediate the Protective Effects of Inactivated Selenium-Enriched Yeast Against Alcohol-Induced Liver Damage in Mice
Source: Foods. 2025 Dec 8;14(24):4209. doi: 10.3390/foods14244209 (PMC12732210; doi:10.3390/foods14244209)
Supplement: Supplementary file 1 [file foods-14-04209-s001.zip › foods-4003670-supplementary.pdf]

Supporting Information

Gut microbiota and liver metabolism regulation mediate the protective effects of inactivated selenium-enriched yeast against alcohol-induced liver damage in mice

Zihua Liang [1,2,†], Xiangchen Zhang [1,2,†], Shiwei Chen [1,2], Meiting Wang [1,2], Deying Men [1,2], Wangxin Liu [1,2] and Xucong Lv [1,2,*]

[1] Institute of Food Science and Technology, College of Biological Science and Technology, Fuzhou University, Fuzhou 350108, China; 2500810007@fzu.edu.cn (Z.L.); 228527202@fzu.edu.cn (X.Z.); 228527170@fzu.edu.cn (S.C.); 2500810011@fzu.edu.cn (M.W.); 240410034@fzu.edu.cn (D.M.); liuwangxin@fzu.edu.cn (W.L.)

[2] Food Nutrition and Health Research Center, School of Advanced Manufacturing, Fuzhou University, Jinjiang 362200, China

* Correspondence: xucong1154@fzu.edu.cn

† These authors contributed equally to this work.

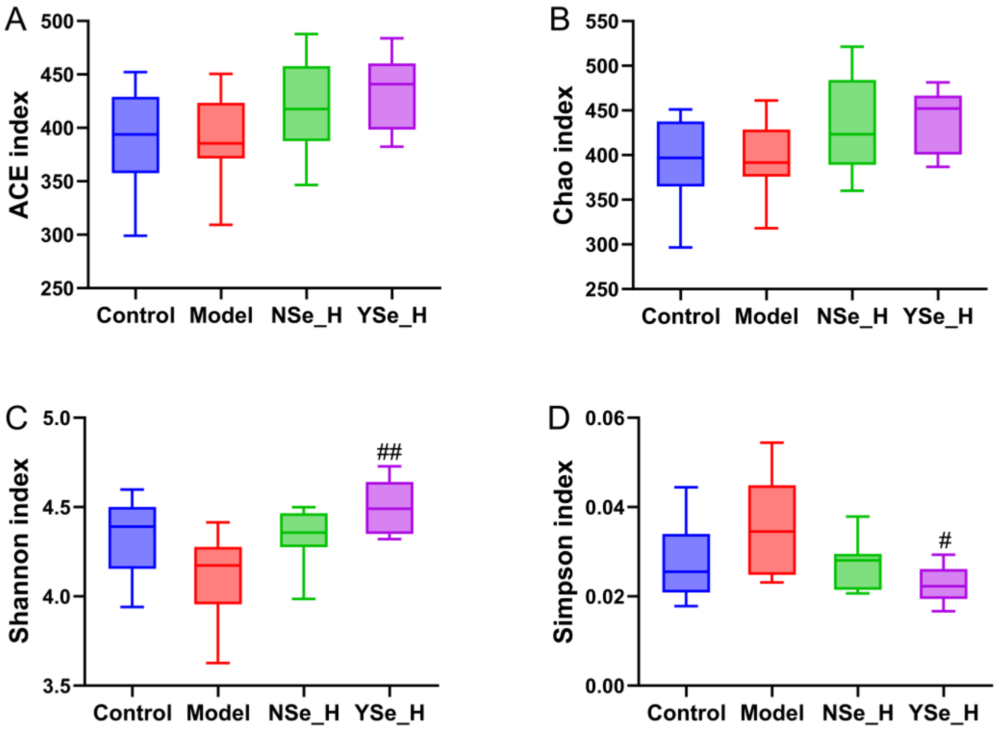

Figure S1. Effects of inactivated YSe and inorganic Se interventions on the alpha diversity of intestinal flora in ALD mice. (A) ACE index. (B) Chao index. (C) Shannon index. (D) Simpson index. Sample sizes: model group, n=10; other groups, n=8. Statistical significance was determined by one-way ANOVA followed by Tukey's post-hoc test. $^{\#}p < 0.05$ and $^{\#\#}p < 0.01$ versus Model group.

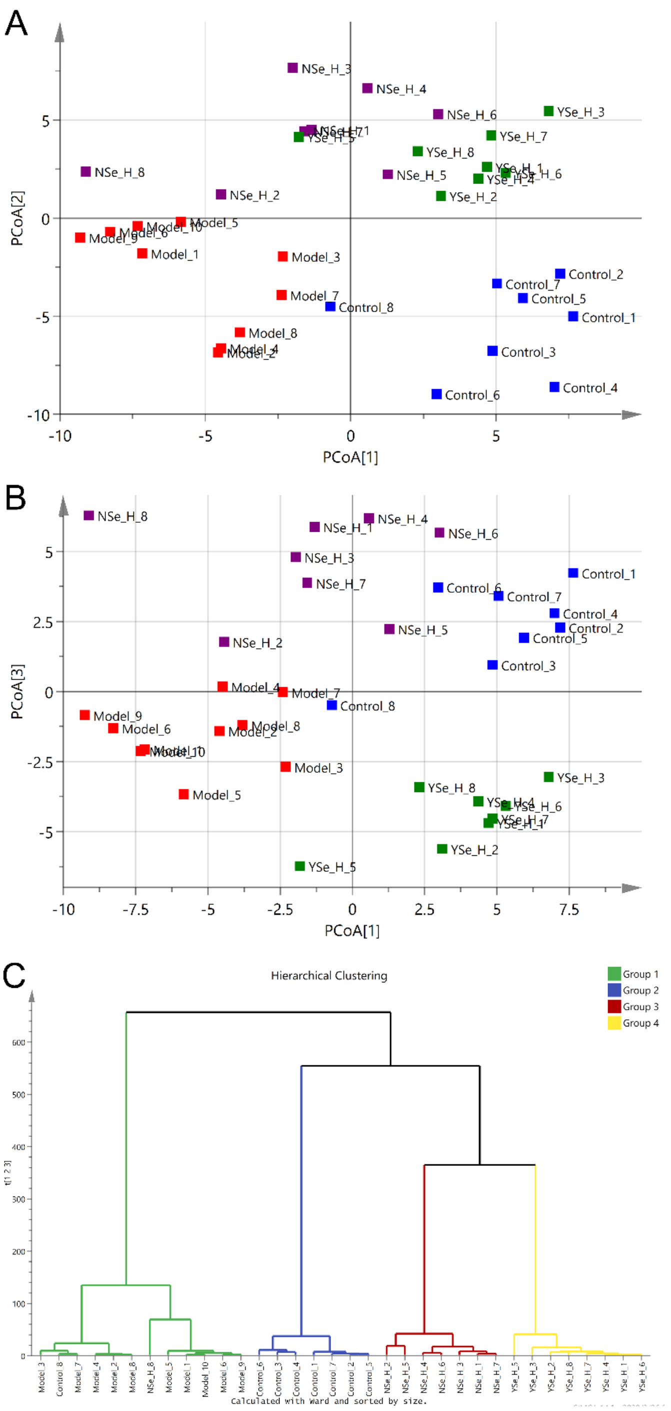

Figure S2. Effects of inactivated YSe and inorganic Se intervention on the composition of intestinal flora in ALD mice at the genus level. (A, B) Principal coordinate analysis (PCoA). (C) Hierarchical cluster analysis diagram. Sample sizes: model group, n=10; other groups, n=8.

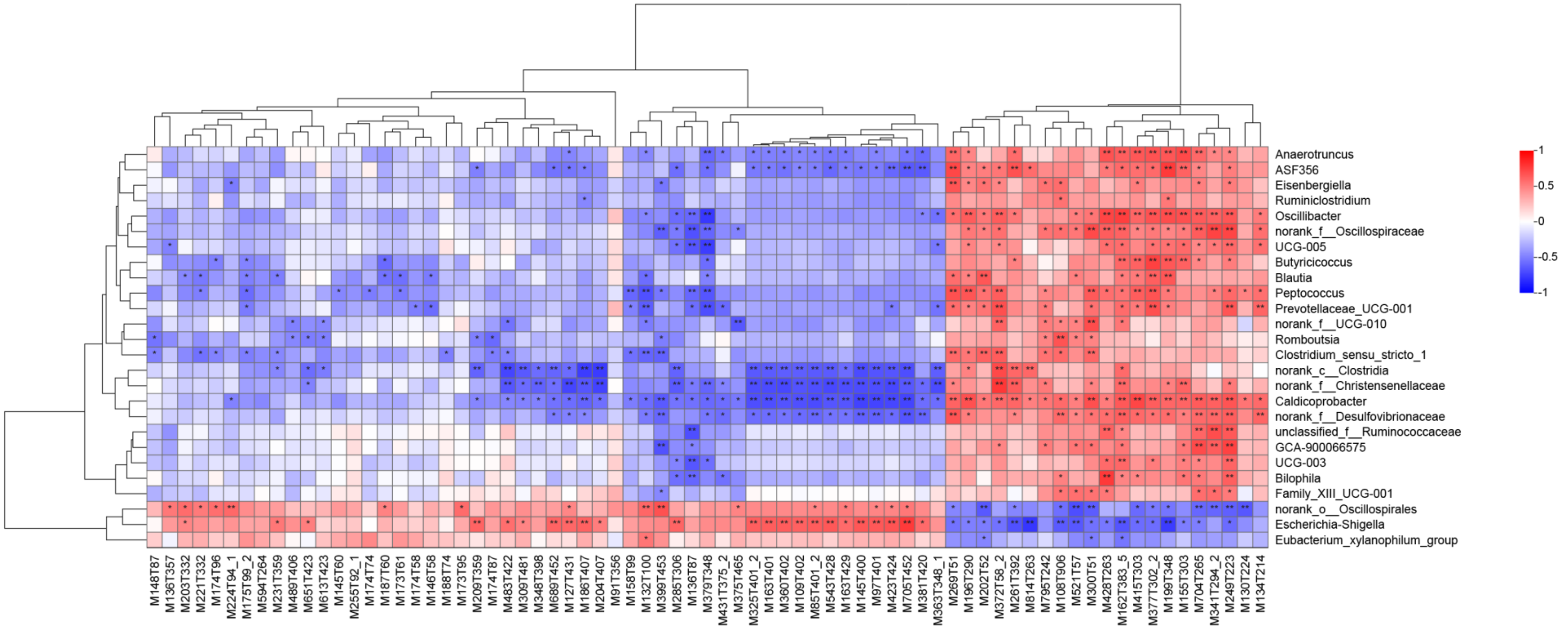

Figure S3. Spearman's Correlation analysis between key intestinal microbes and differential metabolites in the ESI+ mode. Red and blue colors represent positive and negative correlations, respectively. Statistical significance is denoted as *p < 0.05 and **p < 0.01.

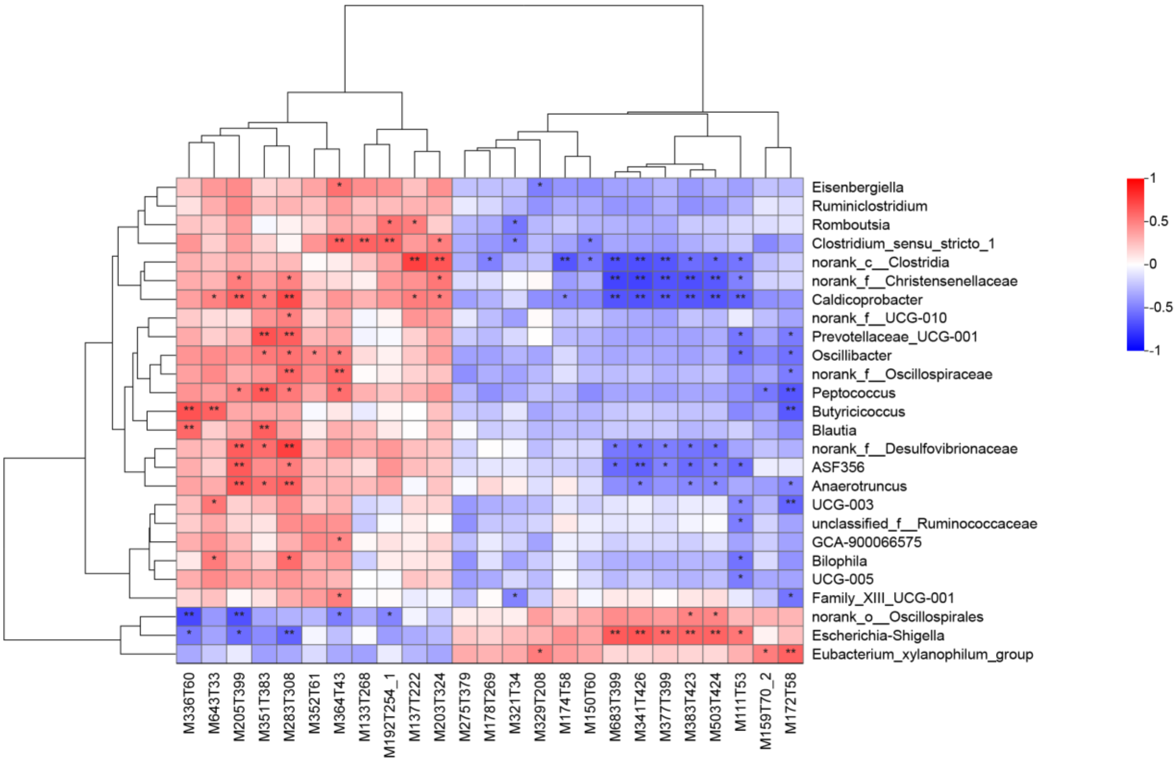

Figure S4. Spearman's Correlation analysis between key intestinal microbes and differential metabolites in the ESI- mode. Red and blue colors represent positive and negative correlations, respectively. Statistical significance is denoted as *$p < 0.05$ and **$p < 0.01$.

Table S1. The abundance of differential metabolites between the Model and YSe-H groups by UPLC-QTOF/MS in ESI+ mode. Differential metabolites between groups were selected based on VIP > 1.0 and $p < 0.05$.

Metabolite ID	Name	HMDB ID	KEGG ID	YSe-H	Model
M224T94_1	Chlorbufam	/	C19060	4.02	87.78
M196T290	D-glucosaminic acid	/	C03752	62.56	9.90
M341T294_2	1-stearoyl-rac-glycerol	/	/	635.19	494.20
M399T453	S-adenosylmethionine	HMDB0001185	C00019	32.41	44.16
M613T423	Glutathione, oxidized	HMDB0003337	C00127	524.05	654.95
M705T452	Laminaritetraose	/	/	154.37	242.48
M136T87	Crimidine	/	C19138	92.41	151.54
M97T401	Methylphosphonic acid	/	C20396	285.44	535.24
M651T423	Caylin-1	/	/	247.31	334.01
M108T906	3-pyridinecarboxaldehyde	/	C07327	63.32	31.52
M174T87	Dihydrorhodamine 123	/	/	52.60	92.78
M145T400	1,6-anhydro-.beta.-d-glucose	HMDB0000640	/	348.08	643.31
M163T401	D-(-)-tagatose	HMDB0003418	C00795	436.91	810.20
M209T359	.alpha.-asarone	HMDB0031469	C17846	144.19	209.72
M300T51	Palmitoyl ethanolamide	HMDB0002100	C16512	442.90	273.85
M85T401_2	1h-1,2,4-triazol-3-amine	/	C11261	546.08	1010.55
M132T100	Zonisamide	HMDB0015045	C07504	61.90	155.25

M375T465	Ile-Asp-Lys	/	/	138.31	171.62
M381T420	Sulfasalazine	HMDB0014933	C07316	32.77	61.55
M221T332	Cys-Val	/	/	71.78	128.02
M325T401_2	Cellobiose	HMDB0000055	C06422	1502.90	2702.22
M377T302_2	(-)-riboflavin	HMDB0000244	C00255	326.74	196.49
M109T402	1,2,3-Benzenetriol	/	/	97.56	176.76
M360T402	Trehalose	HMDB0000975	C01083	2911.23	5254.20
M814T263	N-nervonoyl-d-erythro-sphingosylphosphorylcholine	HMDB0012107	/	191.20	127.75
M372T58_2	Docosahexaenoyl ethanolamide	HMDB0013658	/	166.40	109.24
M173T95	Menadione	HMDB0001892	C05377	27.51	43.96
M285T306	Acacetin	HMDB0132457	C01470	16.31	28.78
M203T332	2-naphthoxyacetic acid	HMDB0032706	C06171	59.62	97.67
M199T348	4-nitrosodiphenylamine	/	C19479	27.72	16.15
M415T303	Isovitexin	/	C01714	51.28	32.89
M423T424	Mangiferin	/	C10077	54.75	146.43
M543T428	Melezitose	HMDB0011730	C08243	646.07	1828.90
M158T99	Indole-3-pyruvic acid	HMDB0060484	C00331	48.79	148.06
M269T51	13-cis-retinol	HMDB0006221	C19962	59.37	30.26
M136T357	Dl-octopamine	/	C04227	1551.17	1881.18
M249T223	Parthenolide	/	C07609	20.56	9.02
M704T265	Palmitoyl sphingomyelin	HMDB0061712	/	1847.57	1397.18

M379T348	Doxorubicin	HMDB0015132	C01661	44.45	67.40
M148T87	Ethyl centralite	/	/	75.13	103.34
M134T214	5-methylbenzotriazole	/	C06473	118.82	45.73
M202T52	Simazine	/	C11172	73.61	51.87
M130T224	.beta.-Homoproline	/	/	41.26	16.44
M594T264	2-(5-oxovaleryl)phosphatidylcholine	/	/	18.40	46.97
M521T57	N-palmitoyl-d-sphingosine	HMDB0004949	/	149.17	114.69
M231T359	Visnagin	/	C09049	33.22	47.64
M795T242	1-(1z-octadecenyl)-2-(5z,8z,11z,14z-eicosatetraenoyl)-sn-glycero-3-phosphocholine	HMDB0011253	/	69.11	48.06
M431T375_2	Bungeiside c	/	/	656.05	883.60
M162T383_5	N-methylephedrine	/	/	92.79	77.25
M174T74	Phosphamidon	/	C18689	72.06	154.28
M175T99_2	L-theanine	HMDB0034365	C01047	462.96	1175.77
M204T407	N-acetyl-d-lactosamine	HMDB0006583	C00611	102.63	130.27
M261T392	Kobusone	/	C16983	103.50	67.54
M127T431	1,3,5-benzenetriol	HMDB0013675	C02183	26.76	45.29
M146T58	4-hydroxyquinoline	/	C06343	87.12	165.18
M428T263	Stearoylcarnitine	HMDB0000848	/	85.70	63.83

* Data were calibrated by the peak area of the internal standard.

Table S2. The abundance of differential metabolites between the Model and YSe-H groups by UPLC-QTOF/MS in ESI- mode. Differential metabolites between groups were selected based on VIP > 1.0 and p < 0.05.

Metabolite ID	Name	HMDB ID	KEGG ID	YSe-H	Model
M205T399	Homocitrate	HMDB0003518	C01251	4.02	87.78
M643T33	Thymidine 5'-monophosphate	HMDB0001227	C00364	62.56	9.90
M329T208	3',5'-cyclic inosine monophosphate	/	/	635.19	494.20
M377T399	Maltose	HMDB0000163	C00208	32.41	44.16
M111T53	Methylphosphonothioic acid o-ethyl ester	/	/	524.05	654.95
M172T58	4-hydroxyquinoline-2-5-carbaldehyde	/	/	154.37	242.48
M321T34	Deoxythymidine 5'-phosphate (dTMP)	HMDB0001227	C00364	92.41	151.54
M336T60	Linoleoylglycine	/	/	285.44	535.24
M150T60	Guanine	HMDB0000132	C00242	247.31	334.01
M192T254_1	Trans-3,5-dimethoxy-4-hydroxycinnamaldehyde	/	C05610	63.32	31.52
M364T43	Amoxicillin	HMDB0015193	C06827	52.60	92.78
M352T61	9h-xanthene-9-propanoic acid	/	/	348.08	643.31
M341T426	1,4-d-xylobiose	/	C01630	436.91	810.20
M683T399	Lactose	HMDB0000186	C00243	144.19	209.72
M503T424	Maltotriose	HMDB0001262	C01835	442.90	273.85

M203T324	D-tryptophan	HMDB0013609	C00806	546.08	1010.55
M383T423	Maltotetraose	HMDB0001296	C02052	61.90	155.25
M178T269	S-carboxymethyl-l-cysteine	HMDB0029415	C03727	138.31	171.62
M283T308	Xanthosine	HMDB0000299	C01762	61.55	32.77
M275T379	6-phosphogluconic acid	HMDB0001316	C00345	71.78	128.02
M351T383	Vitamin c	HMDB0000044	C00072	1502.90	2702.22
M159T70_2	Succinic acid	/	C10996	326.74	196.49
M174T58	N-acetyl-l-aspartic acid	HMDB0000812	C01042	97.56	176.76
M133T268	Malate	HMDB0031518	C00711	2911.23	5254.20
M137T222	3,4-dihydroxybenzaldehyde	HMDB0059965	C16700	191.20	127.75

* Data were calibrated by the peak area of the internal standard.

Table S3. Result from pathway analysis of liver metabolites in ESI+ mode. The Total Cmpd is the total number of compounds in the pathway; the -log10 (p) is obtained by taking the negative logarithm (base 10) of the raw p-value; the FDR p is the p value adjusted using False Discovery Rate; the Impact is the pathway impact value calculated from pathway topology analysis.

Pathway	Total Cmpd	-log10 (p)	FDR p	Impact
Cysteine and methionine metabolism	33	2.913	0.004	0.055
Arginine and proline metabolism	38	2.915	0.004	0.003
Riboflavin metabolism	4	2.291	0.012	0.507
Glutathione metabolism	28	2.031	0.015	0.031
Tryptophan metabolism	41	1.989	0.015	0.001
Starch and sucrose metabolism	15	1.552	0.033	0.001
Glycerophospholipid metabolism	36	1.092	0.080	0.020

Table S4. Result from pathway analysis of liver metabolites in ESI- mode. The Total Cmpd is the total number of compounds in the pathway; the -log10 (p) is obtained by taking the negative logarithm (base 10) of the raw p-value; the FDR p is the p value adjusted using False Discovery Rate; the Impact is the pathway impact value calculated from pathway topology analysis.

Pathway	Total Cmpd	-log10 (p)	FDR p	Impact
Purine metabolism	71	2.521	0.021	0.013
Starch and sucrose metabolism	15	1.422	0.089	0.088
Galactose metabolism	27	1.418	0.089	0.110
Alanine, aspartate and glutamate etabolism	28	0.957	0.166	0.087
Pyrimidine metabolism	39	0.846	0.166	0.087
One carbon pool by folate	26	0.846	0.166	0.009
Pentose phosphate pathway	23	0.411	0.388	0.112
